# The Significance of Kinship for Medical Education: Reflections on the Use of a Bespoke Social Network to Support Learners’ Professional Identities

**DOI:** 10.2196/mededu.4715

**Published:** 2016-03-03

**Authors:** Stylianos Hatzipanagos, Bernadette John, Yuan-Li Tiffany Chiu

**Affiliations:** ^1^ King’s College London Centre for Technology Enhanced Learning King’s College London London United Kingdom

**Keywords:** institutional social networks, collaborative software, medical education, social media

## Abstract

**Background:**

Social media can support and sustain communities much better than previous generations of learning technologies, where institutional barriers undermined any initiatives for embedding formal and informal learning. Some of the many types of social media have already had an impact on student learning, based on empirical evidence. One of these, social networking, has the potential to support communication in formal and informal spaces.

**Objective:**

In this paper we report on the evaluation of an institutional social network—King's Social Harmonisation Project (KINSHIP)—established to foster an improved sense of community, enhance communication, and serve as a space to model digital professionalism for students at King’s College London, United Kingdom.

**Methods:**

Our evaluation focused on a study that examined students’ needs and perceptions with regard to the provision of a cross-university platform. Data were collected from students, including those in the field of health and social care, in order to recommend a practical way forward to address current needs in this area.

**Results:**

The findings indicate that the majority of the respondents were positive about using a social networking platform to develop their professional voice and profiles. Results suggest that timely promotion of the platform, emphasis on interface and learning design, and a clear identity are required in order to gain acceptance as the institutional social networking site.

**Conclusions:**

Empirical findings in this study project an advantage of an institutional social network such a KINSHIP over other social networks (eg, Facebook) because access is limited to staff and students and the site is mainly being used for academic purposes.

## Introduction

Students entering higher education are increasingly digitally literate and capable of using learning technologies, but they still have concerns about technology-enhanced learning replacing face-to-face teaching [1]. Students (both graduate and undergraduate) have varying levels of digital literacy and exposure to social media and associated technologies. Within this context, there is scope for the use of social media to support both formal and informal learning. This paper reports on an exploratory study that examines King’s Social Harmonisation Project (KINSHIP), a social networking platform that meets diverse student needs in health and social care (Medicine and Dentistry) and contributes to the debate on the impact of social networks in higher education.

### Medical Education: a Need for Innovation

Medicine education, with its supporting policies and practices, has a reputation as a conservative discipline, and this is clearly reflected in current/recent educational research [2].

The rather conventional nature of medical education is evidenced by the most common educational practices. Big lecture theater sessions with noninteractive lectures are the dominant practice in many countries (including the United Kingdom). The most frequently used methods of assessing student knowledge are end of term/assessment period examinations and other forms of summative assessment. The hierarchical model of operation can prevent effective interactions and exchange of knowledge between experts and novices. Finally, the lack of links to expert health care professional communities does not support and sustain communities of learners.

On the other hand, research [3] has highlighted aspects of the hidden curriculum for health care practitioners-to-be that are not part of traditional medical educational practice. Leinster [3] points out that clinical and communication skills are common to a range of health care professionals and developing proper attitudes is a major educational goal. Medicine appears to be one of the most appropriate disciplines to become a test bed for alternative educational experiences supported by new learning technologies. These technologies and online social learning, which involve lifelong learners drawing together resources and connections from across the Internet to solve real-life problems often without access to the support of a skilled teacher or accredited learning, are a perfect match for a virtual environment that combines meaningful interaction with realistic challenges [4,5]. Contemporary medical education has been extended to include a variety of digitized learning resources and domain-specific educational activities that offer worldwide access to clinical skills development, independent of time and place [6]. These skills and competencies can be supported or developed by elements of social networking such as Facebook, Second Life, etc [7].

### Social Media and Learning Communities

The rise of social media and social networking has been associated with the transition from content-centered to people-centered activities. Social media sites thrive on the assumption that they operate outside formal learning constraints. Quite often, there is resistance to including the successful characteristics of these sites (eg, visibility, transparency, creation of open communities) in an educational setup [8]. Institutions are also frequently reluctant to allow this out-of-bounds exchange that overrides authentication boundaries commonly associated with an institutional virtual learning environment. Educator confidence in and experience with social media is still perceived as a barrier to successful implementation [9]. In addition, there is limited staff and student awareness of issues of ownership of content and intellectual property rights when using commercial platforms (such as Instagram and Facebook) that assert ownership of user-generated content. This raises concerns for academic discussions on digital platforms, where theories can evolve and intellectual property can be created. Finally, learners are often unwilling to engage in formal, institutional learning interactions in spaces that they consider their own and private [10].

### Student Communities and Social Media: Opportunities

Overall, social media can support and sustain communities much better than previous generations of learning technologies, where institutional barriers undermined initiatives for embedding formal and informal learning. In particular, social media can help users [2,8] achieve enhanced outcomes in relation to more traditional technologies (see Textbox 1).

**Social media potential for learning and teaching [2].**

Social media can help learners to:

- Link to professional communities that can provide feedback, support, and professional identity scaffolding.
- Develop an appropriate, professional digital voice.
- Link to other learner and expert groups, crossing the curriculum horizontally and vertically, so that members are not confined by disciplinary/progression barriers in sharing experiences and learning from others.
- Link to cocurricular and interdisciplinary groups.
- Embed informal and formal lines of communication.
- Create self-help subgroups that can move between boundaries following a “communities of practice” trajectory.
- Embed formal/informal assessment places with an emphasis on formative rather than summative activities.

There are plenty of typologies of social media and a proliferation of tools and services; however, empirical evidence shows that a few key technologies and tools have already had an impact on student learning [11]. Social networking supports communication in formal and informal spaces. Participants in Web-based social networking are immersed in digital environments and engage in acts of computer-mediated communication [12]. Social networking supports virtual communities of people with common interests and exposes articulations of identity [13] through self-representation, performance, and play [14].

## Methods

KINSHIP is a King’s College London social networking site and the deliverable of a King’s College-based project of the same name [15]. The aims of the project were to foster an improved sense of community, enhance communication, and serve as a space to model digital professionalism for students at the university. The original version of KINSHIP was launched in October 2012; further development of functions took place before this spring 2014 pilot.

This case study [16] was conducted employing an online survey (SurveyMonkey) that investigated student views and attitudes toward the site. It targeted all students in the university, including those in health care education (Medicine and Dentistry). A total of 1653 students who registered to use KINSHIP were invited to complete the survey via a link in the circulation email. The survey included multiple choice and open-ended questions for eliciting participant-oriented perspectives. The questions addressed 5 key areas: (1) the relationship between KINSHIP and other social networks, (2) students’ rationales for using KINSHIP, (3) students’ views and attitudes toward the use of KINSHIP (eg, whether they were feeling inhibited about using it, advantages and disadvantages of using KINSHIP, whether the site allowed them to establish a professional digital voice), (4) students’ views on the use of KINSHIP for establishing a sense of academic community, and (5) students’ views on the use of KINSHIP in line with other institutional platforms that supported student learning (eg, the university virtual learning environment).

The open-ended questions were analyzed using NVivo version 10 qualitative software to systematically identify key themes in relation to the students’ views. Only respondents who provided complete responses (ie, answered all questions) were included in our data. In addition, 2 KINSHIP users and medical tutors were interviewed regarding the ability of KINSHIP to support nursing and medical students by putting their skills and knowledge to practice in a health and social care context in undergraduate elective modules. The emerging interview themes were used to complement and triangulate the findings from the survey and helped explore survey themes in depth and gain insights into the use of KINSHIP.

## Results

### Themes From Data Analysis

Several themes were identified from the open-ended survey questions. The following section explores the themes derived from the respondents (67/1653, 4.05%). Where appropriate, we also draw on the interview data concerning the KINSHIP user experiences and perceptions to complement the discussion. The respondents we were interested in had access to social media, with 82.81% (53/64) using a handheld device such as tablet or smartphone and 95.31% (61/64) using a computer. This is interesting in itself, because when KINSHIP was developed initially in 2012, smartphones were not as ubiquitous and social media required logging on at a desktop or laptop. Functionality and accessibility has evolved during the course of the study.

### Theme 1: KINSHIP As an Academic Site to Support Professional Identity

Based on the survey, the majority of respondents (42/65, 64.61%) believed there was merit in the university offering KINSHIP as a space to practice and establish their digital and professional voices.

It is getting more important to use the Internet more selectively and professionally—this is a great way of doing so.

As most students do seem to use social media I think this may be of help in this respect.

Useful for developing a dialogue and professionalism to online debates and/or blogging; useful way to stay linked to societies at King’s without crowding up Facebook.

It helps to keep work/social separate but provide access to a forum where things can be discussed and debated.

**Figure 1:**
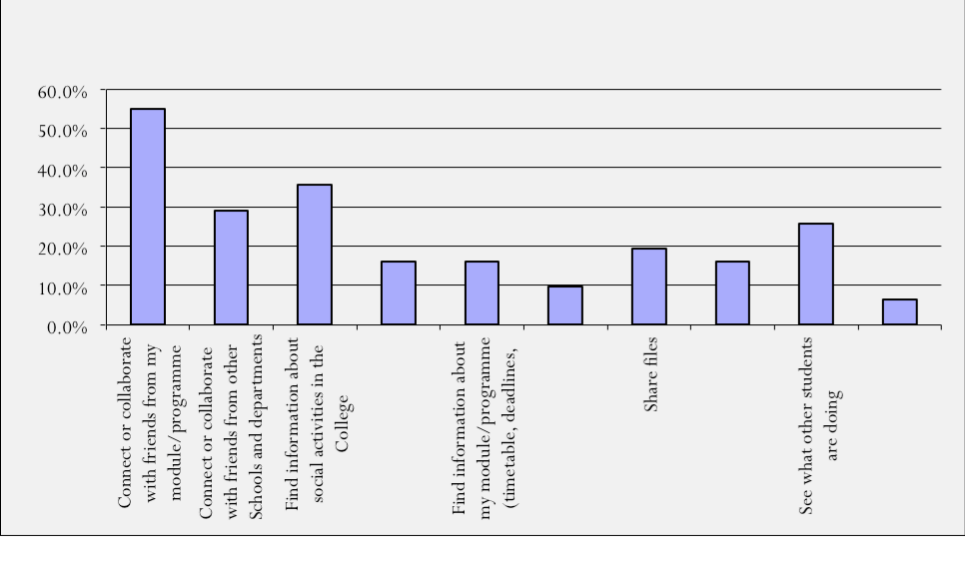
Purpose of using KINSHIP.

The comments above indicate that KINSHIP was beneficial to students for interactions with their peers for academic purposes. In addition, when students were asked to share their thoughts on the purpose of using KINSHIP, a high percentage associated its purpose with academic-related activities (see Figure 1).

As can be seen in Figure 1, the majority of respondents thought that the purpose of using KINSHIP was to connect or collaborate with friends from their modules/program (17/31, 54.83%) and find information about social activities in the university (11/31, 35.48%).

Although the student comments revealed how some of the survey respondents used Facebook for academic purposes such as tutorial groups and assignments (“There is already a Facebook group for my course which I find very useful for this purpose”), such a view is not elicited in the interview data. However, interviewees indicated that KINSHIP provides a suitable platform for activities such as clinical discussions because it is a private, institution-affiliated, social network. A disadvantage of Facebook was the public and permanent nature of what is written on Facebook groups—particularly for clinical students who should not discuss confidential, patient-identifiable information on a commercial platform that can be data mined for income generation purposes.

Many students preferred an institutional networking site like KINSHIP to Facebook because the former is more professional and academically oriented while the latter is primarily used for social purposes.

...I like having the choice to have certain friends on my Facebook. My course requires us to work with other disciplines of health, ie, medical students, dentists, pharmacists, physiotherapists, et cetera. We have to communicate with them, and for the most part social media such as Facebook is the easiest platform. However, I did not particularly want to add them as friends on Facebook for the simple reason being that we were not friends as such. Having KINSHIP gave me the opportunity to use a different social media for work.

The comment above points to an advantage in using KINSHIP for academic purposes because Facebook is mainly geared toward social activities. Students expressed their wish to keep their work and social lives separate.

*I think it is useful for keeping our personal and professional identities separate. While there are other professional media sites, a university-based site also has the potential benefit of providing us with spaces to meet up virtually. This could be particularly useful for tutor groups and interprofessional development activities. I think it also has the potential to be a space to share information and discuss these* [Twitter/blog-style] *with people in our university communities.*

The comment above relates to the use of KINSHIP as a platform for work/academic purposes. When the respondents were asked about using KINSHIP to develop a sense of professional and academic community, more than two-thirds agreed it was a good idea.

You get to know your peers more and would enable you to socialize with more students on the course which may help in study times et cetera.

Useful way to stay linked to societies at King’s without crowding up Facebook.

Useful for society connections and automatically linking people in the same module, firm et cetera.

However, it should also be noted that a high percentage of respondents commented on the importance of KINSHIP uptake when they were asked about the advantages of establishing KINSHIP.

...It’s only useful if lots of people are using/contributing to it which at this stage they aren't, so it's currently not of much benefit to me personally. But I can see that as it grows this may change.

If more people use it, then it could be useful in communicating news and discussing with our peers.

Potentially good as a ‘sandpit.’ The more people who use it, the better it would be.

Yes, but only if the majority of King's students actively use the service.

I suppose that is a good opportunity that KINSHIP can provide, as it is a private network, you can make mistakes without it being accessed globally. Maybe it should be used for courses demonstrating how you can use your social network profile to further your career.

As an academic network this would be extremely useful. More generally, I feel students should be encouraged to be absolutely professional full stop, rather than given the opportunity to voice unprofessional comments in a closed environment.

The above statements echo the interview data. For instance, the medical tutors commented that because the conversations in the KINSHIP forum were mainly associated with the clinical discussion on global health issues, the participating students acted professionally, and the way in which they expressed themselves was also in line with the tone that one could find in the classroom discussions. The tutors stated that conducting this kind of academic collaboration in a network such as KINSHIP may help students develop and establish their professional digital voice. In addition, the tutors made the point that this type of online discussion also facilitates critical reflection and supports global health learning.

### Theme 2: Privacy

When students were asked their opinion on establishing a private social network such as KINSHIP, a majority of respondents were positive as long as it maintained privacy and professionalism standards. Over the course of the study, issues around the security of communications themselves as well as issues around privacy and personal security in general were highlighted in the popular press by the Edward Snowden incident [17]. Respondents voiced concerns about the potential for their communications on KINSHIP being scrutinized by staff, but few had considered the potential for their personal security to be compromised.

Makes it comfortable to discuss confidential issues regarding incidents with patients or problems faced, especially when you don't know who to go to for help.

The interview data highlighted the importance of privacy in clinical discussions in medicine. When the interviewees were asked about their reasons for choosing KINSHIP as a platform for synchronous clinical discussions, they commented:

*Certainly students’ need...was to have a synchronized discussion and to be able to post resources. We have some basic...resources that we were using in the discussions...Most of all with this sort of private social network,...the content would not have the risk of leaking into the public spaces of the Internet...there were still professional discussions as part of the students’ education so we thought that having* [this kind] *of security and privacy was good.... Also, KINSHIP was affiliated with King’s.*

This account points to an important issue that relates to privacy and patient confidentiality, especially in clinical discussions where confidential information needs to be protected. It was indicated that Facebook might not be an ideal platform for such discussions because it is a commercial network that could be nonsecure.

I don't use Facebook. I am concerned about security, privacy, and ownership of content.

This account shows student awareness of privacy and issues around patient confidentiality in online social networks. However, another key privacy issue that came up was concern about monitoring by the university. When responding to the question regarding their inhibitions in using KINSHIP, some respondents expressed privacy concerns.

As it is hosted by King’s, there is a worry that the site is being monitored or tapped into.

Yes, but this is a forum in which I know I will be scrutinized, at least with FB I can be myself separate from my colleagues.

I can carefully control my Facebook privacy settings, as KINSHIP is run by the university I would be reluctant to use something which blurs the boundary between academic/professional and social, in particular with medicine where there should be clear boundary between our work and social side.

The account above reveals that students may fear that their online activities will be scrutinized by staff. However, such an issue was not reflected in the interview data. When the interviewees were asked whether the student users felt inhibited in using KINSHIP, one of them commented:

...They saw us as facilitators so they knew we were reading what they wrote and they also knew what they wrote would be sort of part of this research so they have to consent...There was not a lot sort of personal bits in terms of you know I went here for dinnerFor all the students we had, this was their introduction to KINSHIP so none of them had used KINSHIP previously to this programme...

The response above relates to the nature of the discussion itself, which had an academic orientation. As such, the aspect of feeling inhibited in using KINSHIP may not be perceived as a salient issue in such a context. These issues have evolved and become more important to members over the life of the project

### Theme 3: Visibility and Awareness of KINSHIP

According to the survey data, the KINSHIP platform was unknown to some of the students participating in this pilot. In addition, some of those who were registered stated that they seldom used it.

None of my friends use it.

I joined but have never used it. I do not see its purpose really.

Poor uptake, nobody uses it consistently.

The students’ comments above indicate an important issue that relates to visibility and awareness of social networking sites. These comments may also echo the low response rate in the survey, meaning that a large number of students registered to use KINSHIP, but a smaller number interacted with the site. Promotion and advertisement were recommended to gain a critical mass of users for success. For example, one of the students suggested establishing some events (eg, *KINSHIP Day*) to publicize the site. Students made other recommendations to resolve the issue of low uptake.

Provided it kicks off well and many students get interested in using it, I believe it could be one of the key attraction points of the King’s community. It can provide a great social background for all kinds of people wanting to get to know people on their course or even beyond!

Need to increase membership by encouraging people to use it for university internal socializing and sharing.

It might get more use if it was integrated with existing group work.

KINSHIP was integrated into the Interprofessional education course for all clinical students during the first year of the program, but due to uncertainty with regard to its adoption, this was stopped in the second year. The integration had a clear impact on traffic.

More publicity for it, to get more people regularly using it. Maybe if it had more useful data on it.

Encourage things such as assessment feedback or course information to be continually updated on there. This should be closely updated to ensure it is a good source of information. Make threads which would be engaging for students such as feedback regarding course structure et cetera just to start people off talking.

Perhaps set up an event such as KINSHIP Day in which students from across the university are invited to all sign in on KINSHIP and get to meet people across the university, basically a social day starting say at 6pm where people will all get chatting on KINSHIP. Things could be set up in advance and advertised well to ensure it's successful.

### Theme 4: Facebook As a Point of Reference

When the students were asked about the differences between KINSHIP and established public social networks such as Facebook, Instagram, Snapchat, and WhatsApp, they favored the commercial sites over KINSHIP with respect to interface design, including popularity, ease of access, convenience, functionality, and searchability. This might be related to familiarity of the users with the Facebook interface in relation to a new interface and functionality that KINSHIP represented for them.

One key point raised by the respondents was the lack of a system of notifications of activity via email/instant messaging for KINSHIP, and there was a consensus that KINSHIP should have offered this functionality (this type of functionality became mainstream on commercial platforms shortly after the initial launch of KINSHIP). Another key point from the student responses was user uptake. Specifically, compared with commercial social networking platforms, KINSHIP had a lower user uptake. In comparison, Facebook is virtually ubiquitous. According to the student responses, Facebook has a fast search facility to find people and topics. While considering the type of networking that KINSHIP can offer, many students expressed the view that Facebook is universal (open to everybody) and used for social purposes, whereas KINSHIP was used mainly for academic purposes and restricted to the university student body, an attribute of the KINSHIP design which could alienate some users.

Facebook is more user-friendly; it's not associated with any particular institution.

The main difference is that KINSHIP isn't as inclusive as Facebook is.

Facebook has a much better interface, it's simpler and it has a wide user base already. It's simple to transfer and share files or make public announcements. It's easy to find people. It's fast, it's ubiquitous, and it's easy to search for groups and content.

### Theme 5: KINSHIP’s Interface Design and Ease of Access

Many respondents expressed their dissatisfaction with the existing design/interface of KINSHIP.

You can easily access all of the things you'd like to in an intuitive way through Facebook but not through KINSHIP, where the whole interface feels clunky.

It isn't as spontaneous as Facebook, and the layout is not very appealing.

The student accounts above indicate that the interface design of KINSHIP could benefit from some improvement. Some students also thought that KINSHIP had a limited target audience and was only used for the university student body whereas platforms such as Facebook, Twitter, Instagram, and WhatsApp have a much wider access to other users.

You are more likely to keep in touch with someone on Facebook than KINSHIP. Everyone uses Facebook. KINSHIP is only for university students. I only use one social media [tool], which is Facebook. Twitter and Instagram are being used a lot more now too but Facebook still has the majority of members.

Facebook is full of my friends from outside university as well as university so I never really bothered with KINSHIP as I already have Facebook.

The students seemed to prefer Facebook as the online platform of choice for communication because it allows users to interact with people both within and outside the university whereas KINSHIP is only designed for King’s people.

## Discussion

### Principal Findings

Our findings indicate that the majority of the respondents were positive about using KINSHIP to develop their profile and professional voice. Targeting institutional users and using the platform for a mixture of the formal and the informal have been essential KINSHIP learning design characteristics. Users responded favorably to the separation of purely social interactions and any academic informal or semiformal interactions that KINSHIP can provide.

However, KINSHIP requires a stronger identity to build its niche among other popular social networking platforms. Another recommendation in the survey was that KINSHIP could also become available to other groups of users, such as the university’s alumni. *“It should be heavily used by alumni to make connections. It should be incorporated in the alumni network*” to make connections between past and current students and build professional communities through which uptake could be increased.

In response to student feedback from our data, it is recommended that institutional networking platforms must deliver both in terms of access and interface design. Based on the data, it is also recommended that a networking site may get more use if it is integrated with other institutional tools that students use frequently such as the institutional virtual learning environment or communication platforms. A system of notifications of activities by email, such as daily or weekly digests, was suggested because users may not want to check traffic of communication continuously and directly on the environment.

The empirical evidence of our evaluation points toward a set of interface and learning design specifications that should be an integral part of the design of an institutional social networking site:

1. A well-articulated identity for the platform to achieve wider adoption within the institution.
2. Development and evolution of functionality in step with commercial platforms to achieve a smooth experience for the users who have experience using these external sites.
3. Improving access and redeveloping the social network as a mobile application to benefit from current and ongoing advances in mobile technologies.
4. Addressing privacy concerns raised by students about potential monitoring by staff/institution.
5. Raising awareness about issues around intellectual property ownership and the risks of allowing confidential data to be mined by advertisers when using a commercial platform.
6. Active and consistent promotion to students and staff in order to ensure traffic.
7. Simple authentication process and full integration with the institutional virtual learning environment.
8. Support to students by tutors and moderators when establishing their digital voice.

### Conclusions

This case study explored the specifics of the deployment of a custom-made social network. The results of this study indicate that institutional social networks such as KINSHIP have potential for supporting formal and informal learning. Teaching and learning in medicine can be social and informal and in this context employing such technologies can really help [10]. Specifically, medical student respondents considered KINSHIP a potentially useful space for them to discuss sensitive clinical issues in relation to their work and studies; such a view was also evident in the interview data we analyzed.

The survey we administered to the registered users of the institutional network, despite the relatively low response, provided a rich set of qualitative data. The empirical findings projected an advantage of KINSHIP over other social networks, because it was exclusively targeting people from the university and mainly being used for academic purposes. This is also supported by the interview data of experiences using KINSHIP for online learning for nursing and medical students. Given the nature and purpose of the activities conducted, KINSHIP offers a space for students to interact with colleagues and tutors; it can also be argued that social networking platforms may give a voice to users and help them to develop their digital identity on a personal and professional level.

The richness of the qualitative aspect of our data has given us confidence in our findings, and our experience points toward outcomes that are generalizable, especially the design recommendations. However, further research would be required to explore the adoption of such systems in higher education, and the approach would benefit from looking at comparative studies in different institutions to explore successful adoption and features. The privacy issue raised by students expressing concern about potential monitoring by staff/institution should be investigated.

Institutional networks need promotion to create awareness and knowledge of their existence among student target audiences in order to succeed. The importance of interface design embedded within a learning design narrative was also brought up by the respondents of this study. Another issue which deserves attention was a need to establish a unique identity for these sites as institutional social networks because they are in competition with existing well-established commercial social networks such as Facebook and LinkedIn. An important theme that has also emerged from our study is the perceived importance of privacy; students raised the issue that the degree of privacy protection in KINSHIP requires further elaboration. Institutional policies should address such privacy issues.

Overall, ongoing development of functionality, improved ease of access via redeveloping the platform as a mobile application, and addressing privacy concerns raised by the students about potential monitoring by staff or the institution would be essential if an institutional social networking platform were to be a success.

## Abbreviations

KINSHIP: King's Social Harmonisation Project
